# Supplementary material for: A blueprint for biomolecular condensation driven by bacterial microcompartment encapsulation peptides
Source: Nat Commun. 2025 Aug 11;16:7378. doi: 10.1038/s41467-025-62772-0 (PMC12339742; doi:10.1038/s41467-025-62772-0)
Supplement: Supplementary file 1 — Supplementary Information [file 41467_2025_62772_MOESM1_ESM.pdf]

A blueprint for biomolecular condensation driven by bacterial microcompartment encapsulation peptides

Daniel S. Trettel[1], Cesar A. López[2], Eliana Rodriguez[1], Babetta L. Marrone[1], Cesar Raul Gonzalez-Esquer[1,*]

[1]Los Alamos National Laboratory, Bioscience Division, Microbial and Biome Sciences Group
[2]Los Alamos National Laboratory, Theoretical Biology and Biophysics Group
*Corresponding author: crge@lanl.gov

Supplemental Data

Supplemental Figures 1-11

Supplemental Tables 1-3

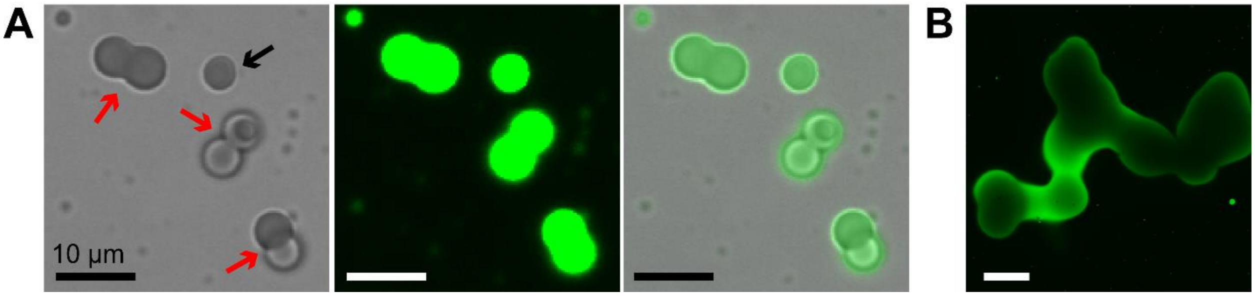

Supplemental Figure 1: Arrested droplet fusion events. **(A)** Droplets were commonly observed in mid-fusion events (red arrows) along with fully septated droplets (black arrow). **(B)** Order of mixing was critical. Adding protein last from a stock solution can lead to large aggregates that appear to be fusions of multiple droplets that could not be kinetically resolved.

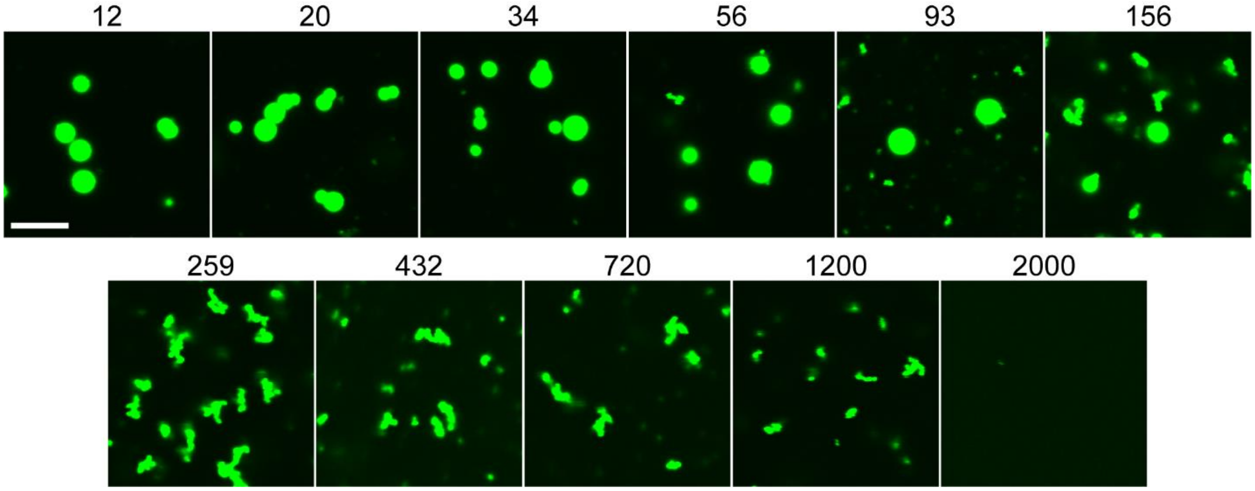

Supplemental Figure 2: Effect of NaCl on EP condensation. Condensation was triggered in the presence of NaCl (indicated above images, in micromolar), as in Figure 3C, and then imaged with a laser scanning confocal microscope. Condensates form cleanly in lower NaCl, eventually reorganizing into aggregates and fully dissolve with sufficient salt. Scale bar, 5 μm.

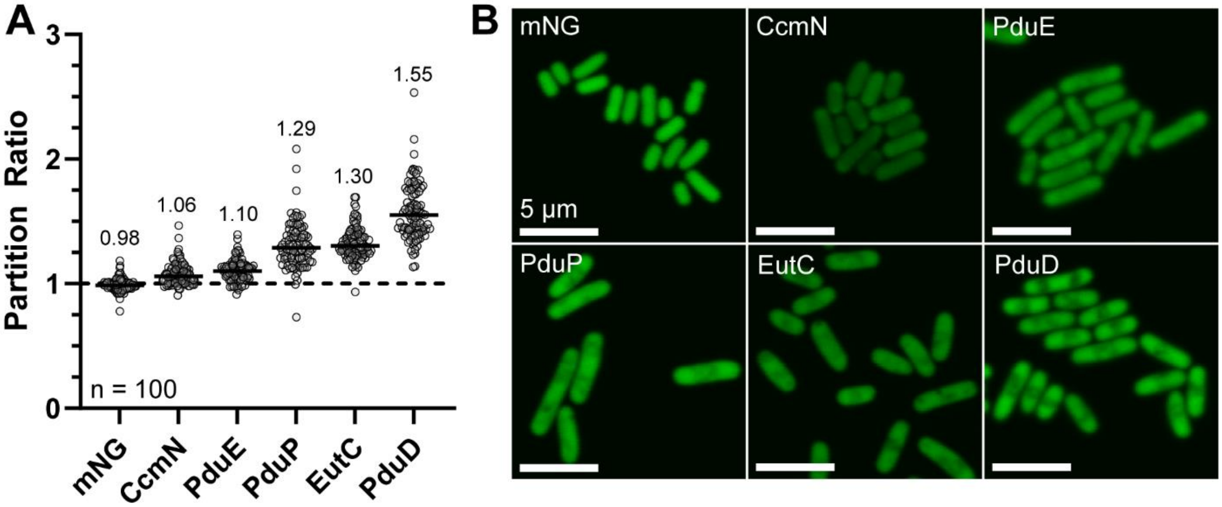

Supplemental Figure 3: Morphological and functional differences for different encapsulation peptides. **(A)** The partition ratio for apparent foci were calculated in cells overexpressing EP-mNeonGreen fusions. These correlate with turbidity data shown in Figure 4 in the main text. The partition ratio was calculated as the intensity of background subtracted foci divided by non-foci areas of the same cell) which is analogous to how much denser foci are compared to the dilute areas of the cell (Figure 6C). **(B)** Cells visually show variances in partitioning, with PduD being the strongest.

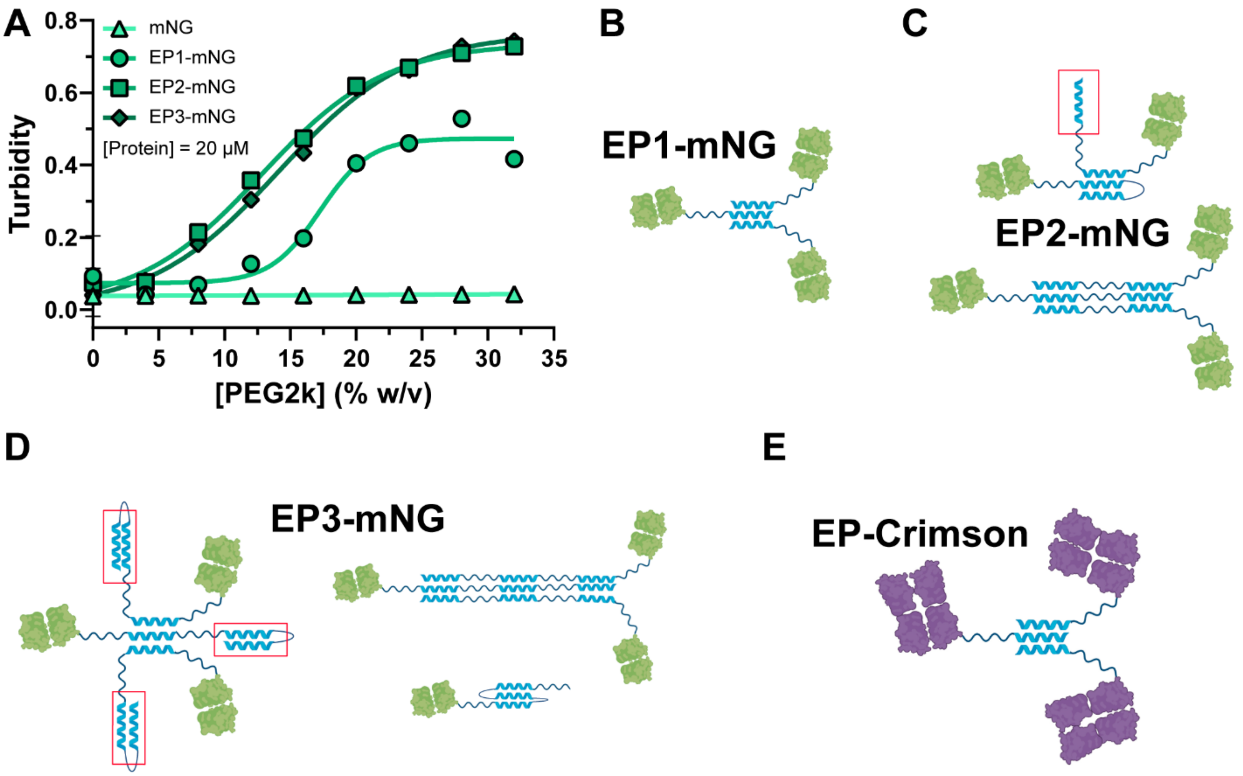

<u>Supplemental Figure 4: Serial additions of EPs lead to diminishing returns for enhancing condensation.</u> **(A)** As when titrating overall protein concentration (Figure 5A in the main text), the EP2 and EP3 designs require less PEG2k to trigger robust biomolecular condensation compared to the EP1 designs. However, the EP3 does not outperform the EP2 design, signifying self-quenching of additional networking. Error bars represent the standard deviation from the mean from 4 replicates **(B-E)** The possible outcomes of EP self-assembly are graphical represented. Red boxes indicate EP bundles with additional binding capacity.

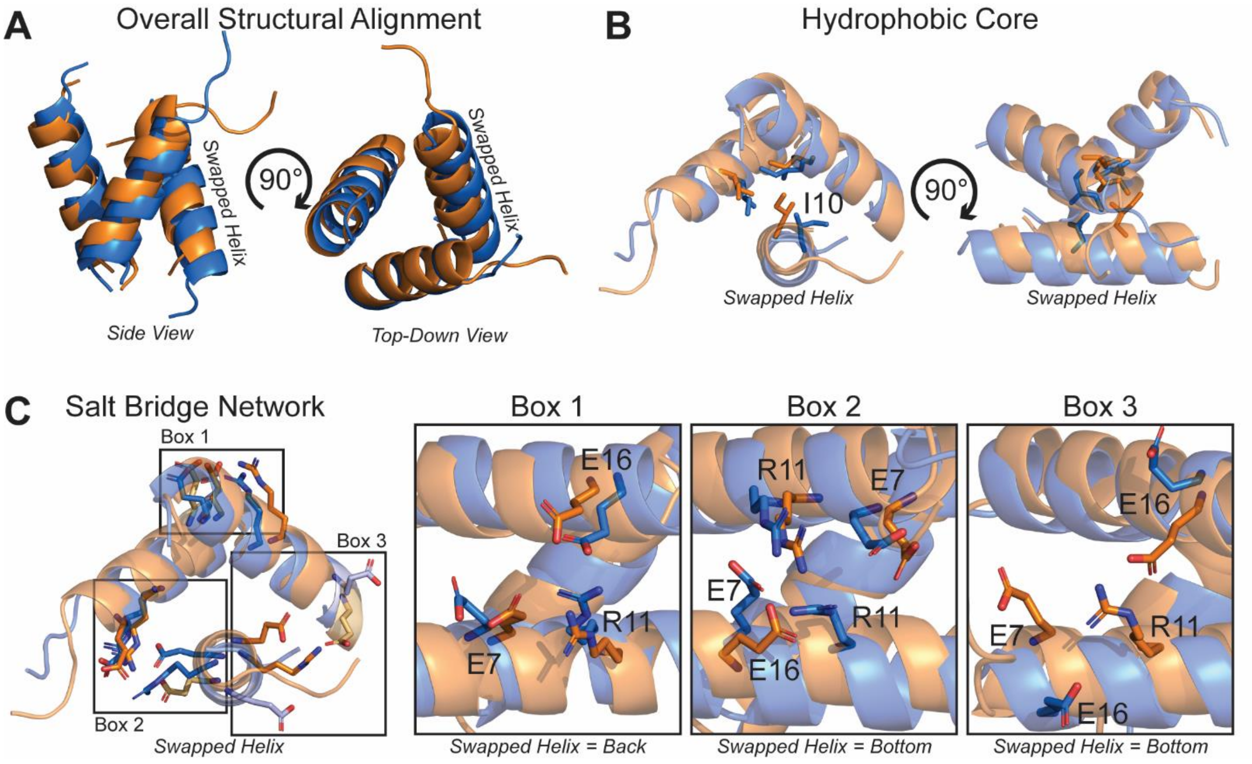

<u>Supplemental Figure 5: Structural comparison of parallel and antiparallel PduP EP trimer bundles.</u> **(A)** Parallel (orange) and antiparallel (blue) configurations of structurally aligned trimeric EP bundles. Only one helix has its orientation swapped between the two configurations. **(B)** Key hydrophobic core residues like I10 are largely positioned similarly between the two configurations. **(C)** Key residues (E7, R11, and E16) in stabilizing salt bridge interactions can form in either configuration. These interactions are grouped into three sets depending on the set of helices forming the interaction surface (labeled boxes). Box 1 shows high conservation of interactions due to the swapped helix not participating. Box 2 shows an antiparallel salt bridge forming in the antiparallel configuration, meanwhile the parallel orientation shows an interaction between R11 and E16. Lastly, Box 3 shows interactions between R11 and E16 in the parallel configuration, while there are no interacting residues in the antiparallel bundle.

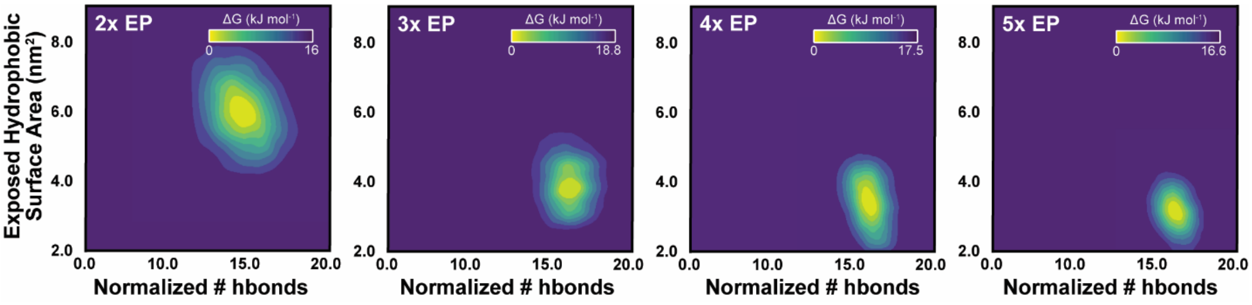

<u>Supplemental Figure 6: Density maps probing the total surface exposure and normalized number of hydrogen bonds.</u> Dimers (2x EP), trimers (3x EP), tetramers (4x EP) and pentamers (5x EP) were assessed.

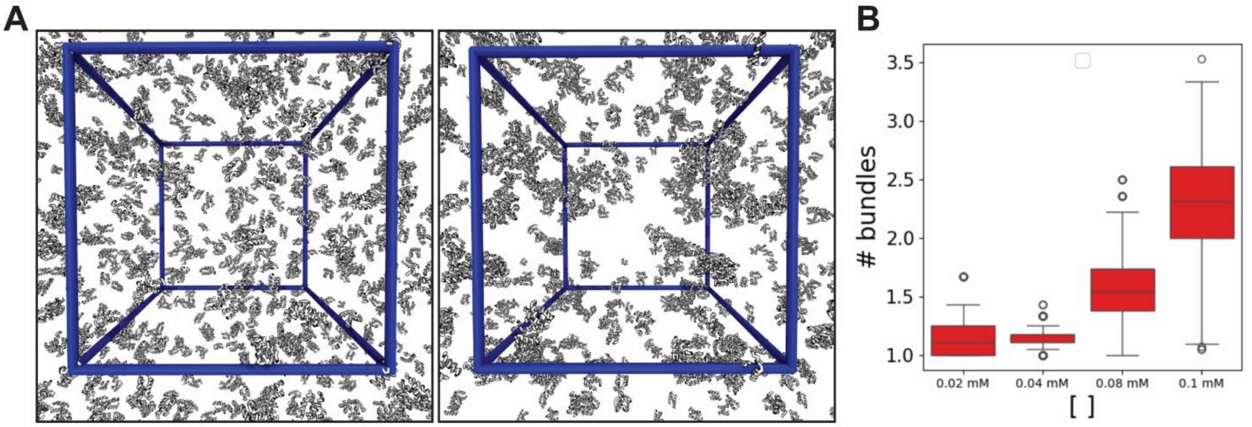

Supplemental Figure 7: Molecular dynamic simulations probe the self-aggregation of trimeric EP bundles. **(A)** System setup of equilibrated trimeric bundles represented with the Martini 3 force field. Left inset initial configuration, right panel equilibrated configuration. Proteins were solvated in an 80/20 water/PEG mixture. **(B)** Average number of associated bundles along the simulation trajectory as a function of protein concentration.

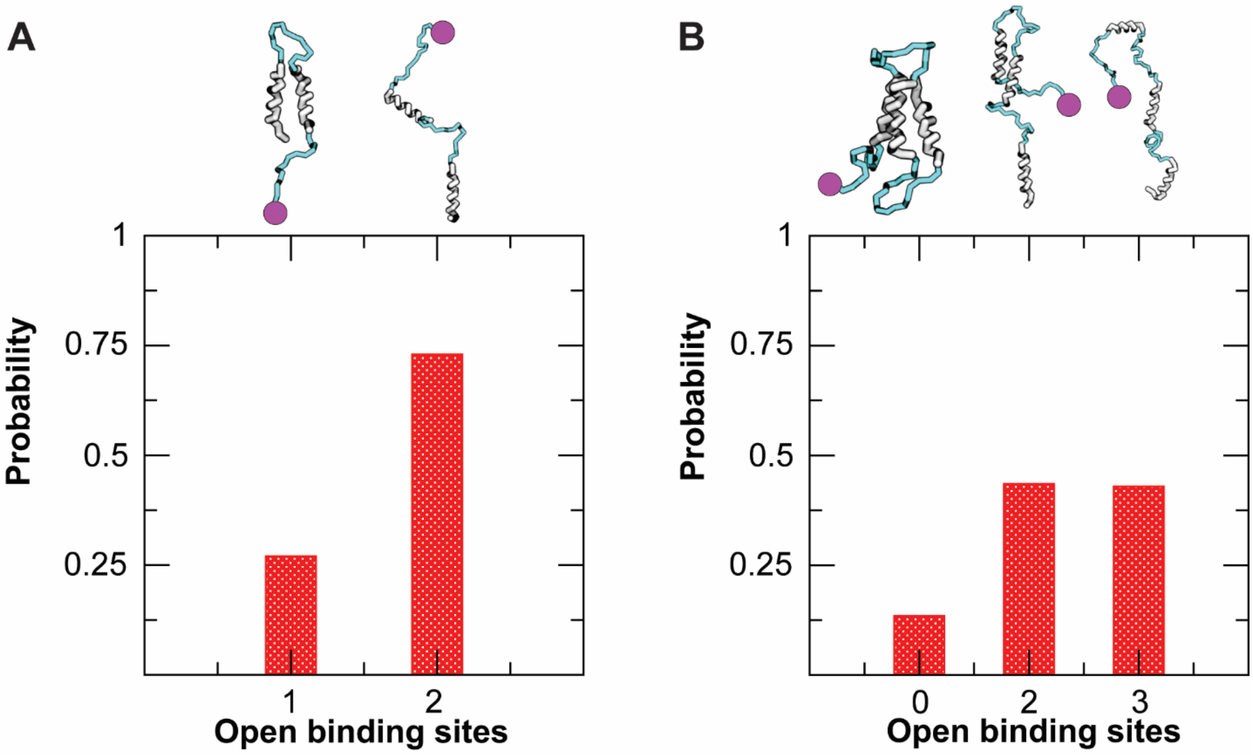

Supplemental Figure 8: EP constructs self-quenching. **(A)** Configuration probability for EP2 construct probed in our molecular dynamic simulations. Insets show typical configurations highlighting the maximum binding capacity. (B) Identical as panel A but for EP3. Magenta sphere represents the attachment region for mNeonGreen.

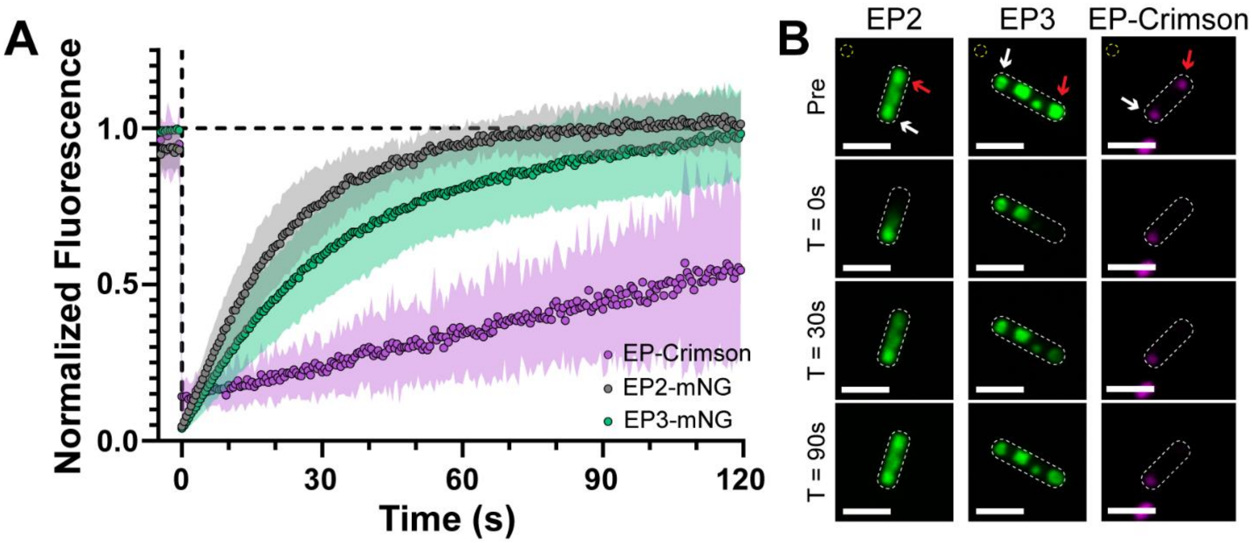

Supplemental Figure 9: Differences in material state for encapsulation peptide fused cargo depending on valency topology. (A) Remake of Figure 6D in the main text with recovery data for the EP-Crimson (emergently valent) design overlays, showing a linear recovery profile compared to the mNeonGreen designs (intrinsically valent). Standard deviations are represented by the infill from 5 replicates. **(B)** This recovery is demonstrated visually by limited reappearance of the bleached foci (red arrow). Scale bar, 1 μm.

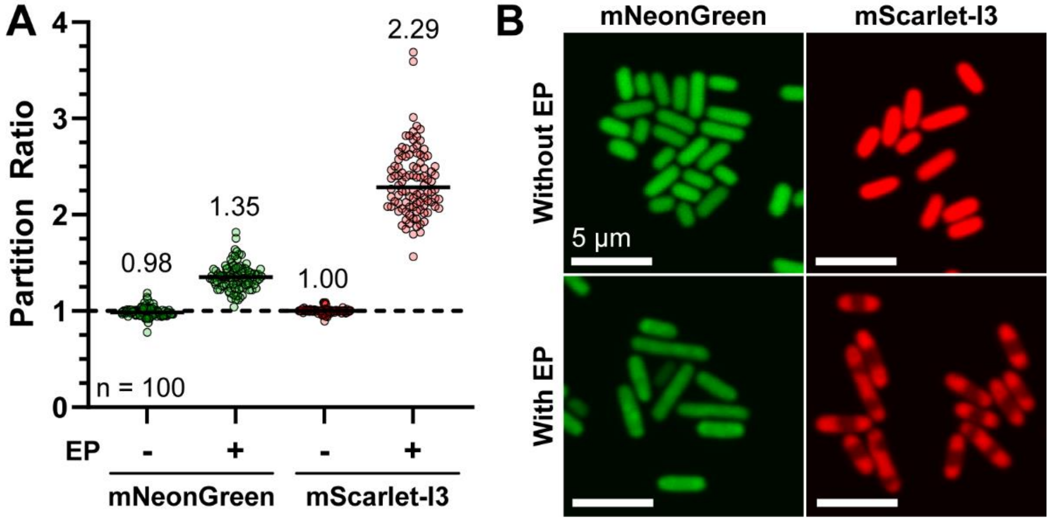

<u>Supplemental Figure 10: Cargo effects on partitioning propensity.</u> (A) An encapsulation peptide fused to mScarlet-I3 resulted in greater partitioning than the same design fused to mNeonGreen. **(B)** Confocal imaging of cells overexpressing cargo fusions showed the greater partitioning ability of mScarlet-I3 *in vivo*.

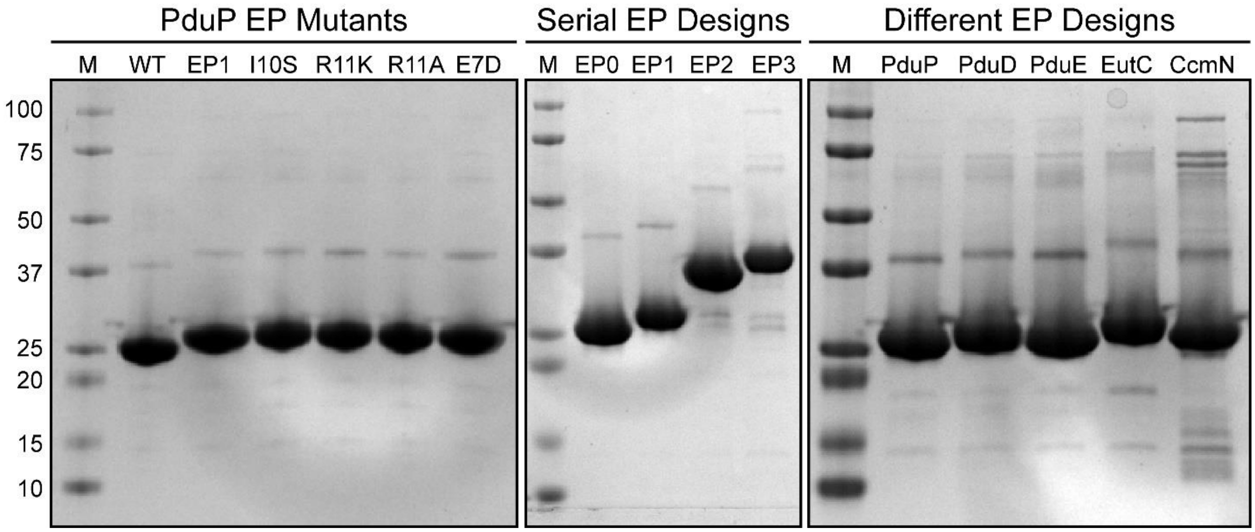

Supplemental Figure 11: SDS-PAGE profiles of purified proteins used in this study.

Supplemental Table 1: List of plasmids used in this study. Acronyms used: G (mNeonGreen with C-terminal 6xHis tag), R (mScarlet-I3), and B (mTagBFP2). Subscripts refer to the number of PduP EP domains fused to the N-terminus.

Name	Source	Genes Encoded	Purpose
pET11a	Novagen (#69436)	None	Vector backbone
pDTV001	This study	G_0	Expression/purification
pDTV002	This study	R_0	Expression/purification
pDTV004	This study	G_1	Expression/purification
pDTV005	This study	R_1	Expression/purification
pDTV007	This study	G_2	Expression/purification
pDTV008	This study	G_3	Expression/purification
pDTV009	This study	$G_0R_0B_0$	Expression/purification
pDTV010	This study	$G_1R_0B_0$	Expression/purification
pDTV016	This study	$G_2R_0B_0$	Expression/purification
pDTV019	This study	$G_1R_1B_1$	Expression/purification
pDTV026	This study	$G_3R_0B_0$	Expression/purification
pDTV056	This study	$G_2R_2B_0$	Expression/purification
pDTV081	This study	R_0-6xHis	Expression/purification
pDTV082	This study	R_1-6xHis	Expression/purification
pDT070	This study	E2-Crimson-6xHis	Expression/purification
pDT071	This study	EP-E2-Crimson-6xHis	Expression/purification
pDT101	This study	I10S G_1	Expression/purification
pDT102	This study	R11K G_1	Expression/purification
pDT103	This study	R11A G_1	Expression/purification
pDT104	This study	E7D G_1	Expression/purification
pDT107	This study	PduD EP-mNeonGreen	Expression/purification
pDT108	This study	PduE EP-mNeonGreen	Expression/purification
pDT109	This study	EutC EP-mNeonGreen	Expression/purification
pDT110	This study	CcmN EP-mNeonGreen	Expression/purification
pDT193	This study	E7R/R11E G_1	Expression/purification

Supplemental Table 2: List of primers used in this study. All primers were ordered from IDT with standard desalting.

Name	Sequence (5'->3')	Purpose
oDT122	CGAATTCGCTAGCCCAAAAAGTTAAACAAAATTATTTCTAGAGGGGAATTG	Linearize pET11a
oDT123	ATGAGAGAAGATTTTCAGCCTGACTGAGCAATAACTAGCATAACCC	Linearize pET11a
oDT168	cagggtttcCAGttcGCTg	Make I10S mutation
oDT169	AGCcgcaccattctgagcGAAC	Make I10S mutation
oDT170	aatcagggtttcCAGttcGC	Make R11K mutation
oDT171	AAAaccattctgagcGAACAGc	Make R11K mutation
oDT172	GCGaccattctgagcGAACAGc	Make R11A mutation
oDT173	CAGttcGCTggtGTTcatttag	Make E7D mutation
oDT174	GATaccctgattcgcaccattc	Make E7D mutation

Supplemental Table 3: List of gene fragments used in this study. All gene fragments were ordered from Twist Biosciences with adaptors off. Underlined sequences are the ribosomal binding site (BBa_B0034), highlighted are the gene reading frames, and bolded are the Gibson assembly overlap regions. Blue bolded overlaps will overlap directly with pET11a when linearized with oDT122 and oDT123, while red and green bolded overlaps are compatible. Fragments were designed for either single (S) assembly or multicomponent (M) assembly.

Name	Gene Encoded	Sequence (5'->3')
gDT022	G_0 (S)	**TTTTTGGGCTAGCGAATTCG**tactagagaaagaggagaaatactaaATGGTGAGCAAAGGC<br>GAAGAAGATAACATGGCGAGCCTGCCGGCGACCCATGAACTGCATATTTTTGGCA<br>GCATTAACGGCGTGGATTTTGATATGGTGGGCCAGGGCACCGGCAACCCGAACG<br>ATGGCTATGAAGAACTGAACCTGAAAAGCACCAAAGGCGATCTGCAGTTTAGCCC<br>GTGGATTCTGGTGCCGCATATTGGCTATGGCTTTCATCAGTATCTGCCGTATCCGG<br>ATGGCATGAGCCCGTTTCAGGCGGCGATGGTGGATGGCAGCGGCTATCAGGTG<br>CATCGCACCATGCAGTTTGAAGATGGCGCGAGCCTGACCGTGAACTATCGCTATA<br>CCTATGAAGGCAGCCATATTAAAGGCGAAGCGCAGGTGAAAGGCACCGGCTTTC<br>CGGCGGATGGCCCGGTGATGACCAACAGCCTGACCGCGGCGGATTGGTGCCG<br>CAGCAAAAAAACCTATCCGAACGATAAAACCATTATTAGCACCTTTAAATGGAGCT<br>ATACCACCGGCAACGGCAAACGCTATCGCAGCACCGCGCGCACCACCTATACCT<br>TTGCGAAACCGATGGCGGCGAACTATCTGAAAAACCAGCCGATGTATGTGTTTCG<br>CAAGACCGAACTGAAACATAGCAAAACGGAGCTGAACTTTAAAGAATGGCAGAAA<br>GCGTTTACCGATGTGATGGGCATGGATGAACTGTATAAAGGCGGTAGCGGCGGTA<br>GCCACCATCACCATCACCATTAA**ATGAGAGAAGATTTTCAGCCTGA**
gDT023	R_0 (S)	**TTTTTGGGCTAGCGAATTCG**tactagagaaagaggagaaatactaaATGGATAGCACCGAA<br>GCGGTGATTAAAGAATTTATGCGCTTTAAAGTGCATATGGAAGGCAGCATGAACG<br>GCCATGAATTTGAAATTGAAGGCGAAGGCGAAGGCCGCCCGTATGAAGGCACCC<br>AGACCGCGAAACTGAAAGTGACCAAAGGCGGCCCGCTGCCGTTTAGCTGGGAT<br>ATTCTGAGCCCGCAGTTTATGTATGGCAGCCGCGCGTTTATTAAACATCCGGCGG<br>ATATTCCGGATTATTGGAAACAGAGCTTTCCGGAAGGCTTTAAATGGGAACGCGT<br>GATGATTTTTGAAGATGGCGGCACCGTGAGCGTGACCCAGGATACCAGCCTGGA<br>AGATGGCACCCTGATTTATAAAGTGAAACTGCGCGGCGGCAACTTTCCGCCGGA<br>TGGCCCGGTGATGCAGAAACGCACCATGGGCTGGGAAGCGAGCACCGAACGCC<br>TGTATCCGGAAGATGTGGTGCTGAAAGGCGATATTAAAATGGCGCTGCGCCTGAA<br>AGATGGCGGCCGCTATCTGGCGGATTTTAAAACCACCTATAAAGCGAAAAAACCG<br>GTGCAGATGCCGGGCGCGTTTAACATTGATCGCAAACTGGATATTACCAGCCATA<br>ACGAAGATTATACCGTGGTGGAACAGTATGAACGCAGCGTGGCGCGCCATAGCA<br>CCGGCGGCAGCGGCGGCAGCTAA**ATGAGAGAAGATTTTCAGCCTGA**
gDT025	G_1 (S)	**TTTTTGGGCTAGCGAATTCG**tactagagaaagaggagaaatactaaatgAACaccAGCgaaCTG<br>gaaaccctgattcgcaccattctgagcGAACAGctgaccACCccggcgCAGACCccgGTGcagccgca<br>gggcAAAGGCattTTTcagAGCGTGAGCAAAGGCGAAGAAGATAACATGGCGAGCCT<br>GCCGGCGACCCATGAACTGCATATTTTTGGCAGCATTAACGGCGTGGATTTTGAT<br>ATGGTGGGCCAGGGCACCGGCAACCCGAACGATGGCTATGAAGAACTGAACCT<br>GAAAAGCACCAAAGGCGATCTGCAGTTTAGCCCGTGGATTCTGGTGCCGCATATT<br>GGCTATGGCTTTCATCAGTATCTGCCGTATCCGGATGGCATGAGCCCGTTTCAGG<br>CGGCGATGGTGGATGGCAGCGGCTATCAGGTGCATCGCACCATGCAGTTTGAAG<br>ATGGCGCGAGCCTGACCGTGAACTATCGCTATACCTATGAAGGCAGCCATATTAA<br>AGGCGAAGCGCAGGTGAAAGGCACCGGCTTTCCGGCGGATGGCCCGGTGATGA<br>CCAACAGCCTGACCGCGGCGGATTGGTGCCGCAGCAAAAAAACCTATCCGAAC<br>GATAAAACCATTATTAGCACCTTTAAATGGAGCTATACCACCGGCAACGGCAAACG<br>CTATCGCAGCACCGCGCGCACCACCTATACCTTTGCGAAACCGATGGCGGCGAA<br>CTATCTGAAAAACCAGCCGATGTATGTGTTTCGCAAGACCGAACTGAAACATAGC<br>AAAACGGAGCTGAACTTTAAAGAATGGCAGAAAGCGTTTACCGATGTGATGGGCA<br>TGGATGAACTGTATAAAGGCGGTAGCGGCGGTAGCCACCATCACCATCACCATTA<br>A**ATGAGAGAAGATTTTCAGCCTGA**
gDT026	R_1 (S)	**TTTTTGGGCTAGCGAATTCG**tactagagaaagaggagaaatactaaatgAACaccAGCgaaCTG<br>gaaaccctgattcgcaccattctgagcGAACAGctgaccACCccggcgCAGACCccgGTGcagccgca<br>gggcAAAGGCattTTTcagAGCGATAGCACCGAAGCGGTGATTAAAGAATTTATGCGC<br>TTTAAAGTGCATATGGAAGGCAGCATGAACGGCCATGAATTTGAAATTGAAGGCG

		AAGGCGAAGGCCGCCCGTATGAAGGCACCCAGACCGCGAAACTGAAAGTGACCAAAGGCGGCCCGCTGCCGTTTAGCTGGGATATTCTGAGCCCGCAGTTTATGTATGGCAGCCGCGCGTTTATTAAACATCCGGCGGATATTCCGGATTATTGGAAACAGAGCTTTCCGGAAGGCTTTAAATGGGAACGCGTGATGATTTTTGAAGATGGCGGCACCGTGAGCGTGACCCAGGATACCAGCCTGGAAGATGGCACCCTGATTTATAAAGTGAAACTGCGCGGCGGCAACTTTCCGCCGGATGGCCCGGTGATGCAGAAACGCACCATGGGCTGGGAAGCGAGCACCGAACGCCTGTATCCGGAAGATGTGGTGCTGAAAGGCGATATTAAAATGGCGCTGCGCCTGAAAGATGGCGGCCGCTATCTGGCGGATTTTAAAACCACCTATAAAGCGAAAAAACCGGTGCAGATGCCGGGCGCGTTTAACATTGATCGCAAACTGGATATTACCAGCCATAACGAAGATTATACCGTGGTGGAACAGTATGAACGCAGCGTGGCGCGCCATAGCACCGGCGGCAGCGGCGGCAGCTAA**ATGAGAGAAGATTTTCAGCCTGA**
gDT028	G_2 (S)	**TTTTTGGGCTAGCGAATTCG**tactagagaaagaggagaaatactaaatgAACaccAGCgaaCTGgaaaccctgattcgcaccattctgagcGAACAGctgaccACCccggcgCAGACCccgGTGcagccgcagggcAAAGGCattTTTcagAGCAACacgAGCgaaCTGgagaccctgatccgcacgattctgtcgGAACAActgaccACGccggcgCAAACCcctGTGcagcctcagggtAAAGGCattTTCcagAGCGTGAGCAAAGGCGAAGAAGATAACATGGCGAGCCTGCCGGCGACCCATGAACTGCATATTTTTGGCAGCATTAACGGCGTGGATTTTGATATGGTGGGCCAGGGCACCGGCAACCCGAACGATGGCTATGAAGAACTGAACCTGAAAAGCACCAAAGGCGATCTGCAGTTTAGCCCGTGGATTCTGGTGCCGCATATTGGCTATGGCTTTCATCAGTATCTGCCGTATCCGGATGGCATGAGCCCGTTTCAGGCGGCGATGGTGGATGGCAGCGGCTATCAGGTGCATCGCACCATGCAGTTTGAAGATGGCGCGAGCCTGACCGTGAACTATCGCTATACCTATGAAGGCAGCCATATTAAAGGCGAAGCGCAGGTGAAAGGCACCGGCTTTCCGGCGGATGGCCCGGTGATGACCAACAGCCTGACCGCGGCGGATTGGTGCCGCAGCAAAAAAACCTATCCGAACGATAAAACCATTATTAGCACCTTTAAATGGAGCTATACCACCGGCAACGGCAAACGCTATCGCAGCACCGCGCGCACCACCTATACCTTTGCGAAACCGATGGCGGCGAACTATCTGAAAAACCAGCCGATGTATGTGTTTCGCAAGACCGAACTGAAACATAGCAAAACGGAGCTGAACTTTAAAGAATGGCAGAAAGCGTTTACCGATGTGATGGGCATGGATGAACTGTATAAAGGCGGTAGCGGCGGTAGCCACCATCACCATCACCATTAA**ATGAGAGAAGATTTTCAGCCTGA**
gDT029	G_3 (S)	**TTTTTGGGCTAGCGAATTCG**tactagagaaagaggagaaatactaaatgAACaccAGCgaaCTGgaaaccctgattcgcaccattctgagcGAACAGctgaccACCccggcgCAGACCccgGTGcagccgcagggcAAAGGCattTTTcagAGCAACacgAGCgaaCTGgagaccctgatccgcacgattctgtcgGAACAActgaccACGccggcgCAAACCcctGTGcagcctcagggtAAAGGCattTTCcagAGCAATactAGCgagCTGgagactctgattcgtactattctgtcgGAGCAGctgacgACCccggcaCAGACCccgGTTcagcctcaaggcAAGGGTattTTTcagAGCGTGAGCAAAGGCGAAGAAGATAACATGGCGAGCCTGCCGGCGACCCATGAACTGCATATTTTTGGCAGCATTAACGGCGTGGATTTTGATATGGTGGGCCAGGGCACCGGCAACCCGAACGATGGCTATGAAGAACTGAACCTGAAAAGCACCAAAGGCGATCTGCAGTTTAGCCCGTGGATTCTGGTGCCGCATATTGGCTATGGCTTTCATCAGTATCTGCCGTATCCGGATGGCATGAGCCCGTTTCAGGCGGCGATGGTGGATGGCAGCGGCTATCAGGTGCATCGCACCATGCAGTTTGAAGATGGCGCGAGCCTGACCGTGAACTATCGCTATACCTATGAAGGCAGCCATATTAAAGGCGAAGCGCAGGTGAAAGGCACCGGCTTTCCGGCGGATGGCCCGGTGATGACCAACAGCCTGACCGCGGCGGATTGGTGCCGCAGCAAAAAAACCTATCCGAACGATAAAACCATTATTAGCACCTTTAAATGGAGCTATACCACCGGCAACGGCAAACGCTATCGCAGCACCGCGCGCACCACCTATACCTTTGCGAAACCGATGGCGGCGAACTATCTGAAAAACCAGCCGATGTATGTGTTTCGCAAGACCGAACTGAAACATAGCAAAACGGAGCTGAACTTTAAAGAATGGCAGAAAGCGTTTACCGATGTGATGGGCATGGATGAACTGTATAAAGGCGGTAGCGGCGGTAGCCACCATCACCATCACCATTAA**ATGAGAGAAGATTTTCAGCCTGA**
gDT030	G_0 (M)	**TTTTTGGGCTAGCGAATTCG**tactagagaaagaggagaaatactaaATGGTGAGCAAAGGCGAAGAAGATAACATGGCGAGCCTGCCGGCGACCCATGAACTGCATATTTTTGGCAGCATTAACGGCGTGGATTTTGATATGGTGGGCCAGGGCACCGGCAACCCGAACGATGGCTATGAAGAACTGAACCTGAAAAGCACCAAAGGCGATCTGCAGTTTAGCCCGTGGATTCTGGTGCCGCATATTGGCTATGGCTTTCATCAGTATCTGCCGTATCCGGATGGCATGAGCCCGTTTCAGGCGGCGATGGTGGATGGCAGCGGCTATCAGGTGCATCGCACCATGCAGTTTGAAGATGGCGCGAGCCTGACCGTGAACTATCGCTATACCTATGAAGGCAGCCATATTAAAGGCGAAGCGCAGGTGAAAGGCACCGGCTTTCCGGCGGATGGCCCGGTGATGACCAACAGCCTGACCGCGGCGGATTGGTGCCGCAGCAAAAAAACCTATCCGAACGATAAAACCATTATTAGCACCTTTAAATGGAGCTATACCACCGGCAACGGCAAACGCTATCGCAGCACCGCGCGCACCACCTATACCTTTGCGAAACCGATGGCGGCGAACTATCTGAAAAACCAGCCGATGTATGTGTTTCGCAAGACCGAACTGAAACATAGCAAAACGGAGCTGAACTTTAAAGAATGGCAGAAA

		GCGTTTACCGATGTGATGGGCATGGATGAACTGTATAAAGGCGGTAGCGGCGGTA<br>GCCACCATCACCATCACCATTAA**AGCATAGCACAACGATAGCATT**
gDT031	R_0 (M)	**AGCATAGCACAACGATAGCATT**tactagagaaagaggagaaatactaaATGGATAGCACCG<br>AAGCGGTGATTAAAGAATTTATGCGCTTTAAAGTGCATATGGAAGGCAGCATGAAC<br>GGCCATGAATTTGAAATTGAAGGCGAAGGCGAAGGCCGCCCGTATGAAGGCACC<br>CAGACCGCGAAACTGAAAGTGACCAAAGGCGGCCCGCTGCCGTTTAGCTGGGA<br>TATTCTGAGCCCGCAGTTTATGTATGGCAGCCGCGCGTTTATTAAACATCCGGCG<br>GATATTCCGGATTATTGGAAACAGAGCTTTCCGGAAGGCTTTAAATGGGAACGCG<br>TGATGATTTTTGAAGATGGCGGCACCGTGAGCGTGACCCAGGATACCAGCCTGG<br>AAGATGGCACCCTGATTTATAAAGTGAAACTGCGCGGCGGCAACTTTCCGCCGG<br>ATGGCCCGGTGATGCAGAAACGCACCATGGGCTGGGAAGCGAGCACCGAACGC<br>CTGTATCCGGAAGATGTGGTGCTGAAAGGCGATATTAAAATGGCGCTGCGCCTGA<br>AAGATGGCGGCCGCTATCTGGCGGATTTTAAAACCACCTATAAAGCGAAAAAACC<br>GGTGCAGATGCCGGGCGCGTTTAACATTGATCGCAAACTGGATATTACCAGCCAT<br>AACGAAGATTATACCGTGGTGGAACAGTATGAACGCAGCGTGGCGCGCCATAGC<br>ACCGGCGGCAGCGGCGGCAGCTAA**TCGTTCAGTTTGGCTAACTCAT**
gDT032	B_0 (M)	**TCGTTCAGTTTGGCTAACTCAT**tactagagaaagaggagaaatactaaATGGTGAGCAAAG<br>GCGAAGAACTGATTAAAGAAAACATGCATATGAAACTGTATATGGAAGGCACCGTG<br>GATAACCATCATTTTAAATGCACCAGCGAAGGCGAAGGCAAACCGTATGAAGGCA<br>CCCAGACCATGCGCATTAAAGTGGTGGAAGGCGGCCCGCTGCCGTTTGCGTTTG<br>ATATTCTGGCGACCAGCTTTCTGTATGGCAGCAAAACCTTTATTAACCATACCCAG<br>GGCATTCCGGATTTTTTTAAACAGAGCTTTCCGGAAGGCTTTACCTGGGAACGCG<br>TGACCACCTATGAAGATGGCGGCGTGCTGACCGCGACCCAGGATACCAGCCTGC<br>AGGATGGCTGCCTGATTTATAACGTGAAAATTCGCGGCGTGAACTTTACCAGCAA<br>CGGCCCGGTGATGCAGAAAAAAACCCTGGGCTGGGAAGCGTTTACCGAAACCC<br>TGTATCCGGCGGATGGCGGCCTGGAAGGCCGCAACGATATGGCGCTGAAACTG<br>GTGGGCGGCAGCCATCTGATTGCGAACGCGAAAACCACCTATCGCAGCAAAAAA<br>CCGGCGAAAAACCTGAAAATGCCGGGCGTGTATTATGTGGATTATCGCCTGGAAC<br>GCATTAAAGAAGCGAACAACGAAACCTATGTGGAACAGCATGAAGTGGCGGTGG<br>CGCGCTATTGCGATCTGCCGAGCAAACTGGGCCATAAACTGAACTAA**ATGAGAG**<br>**AAGATTTTCAGCCTGA**
gDT033	G_1 (M)	**TTTTTGGGCTAGCGAATTCG**tactagagaaagaggagaaatactaaatgAACaccAGCgaaCTG<br>gaaaccctgattcgcaccattctgagcGAACAGctgaccACCccggcgCAGACCccgGTGcagccgca<br>gggcAAAGGCattTTTcagAGCGTGAGCAAAGGCGAAGAAGATAACATGGCGAGCCT<br>GCCGGCGACCCATGAACTGCATATTTTTGGCAGCATTAACGGCGTGGATTTTGAT<br>ATGGTGGGCCAGGGCACCGGCAACCCGAACGATGGCTATGAAGAACTGAACCT<br>GAAAAGCACCAAAGGCGATCTGCAGTTTAGCCCGTGGATTCTGGTGCCGCATATT<br>GGCTATGGCTTTCATCAGTATCTGCCGTATCCGGATGGCATGAGCCCGTTTCAGG<br>CGGCGATGGTGGATGGCAGCGGCTATCAGGTGCATCGCACCATGCAGTTTGAAG<br>ATGGCGCGAGCCTGACCGTGAACTATCGCTATACCTATGAAGGCAGCCATATTAA<br>AGGCGAAGCGCAGGTGAAAGGCACCGGCTTTCCGGCGGATGGCCCGGTGATGA<br>CCAACAGCCTGACCGCGGCGGATTGGTGCCGCAGCAAAAAAACCTATCCGAAC<br>GATAAAACCATTATTAGCACCTTTAAATGGAGCTATACCACCGGCAACGGCAAACG<br>CTATCGCAGCACCGCGCGCACCACCTATACCTTTGCGAAACCGATGGCGGCGAA<br>CTATCTGAAAAACCAGCCGATGTATGTGTTTCGCAAGACCGAACTGAAACATAGC<br>AAAACGGAGCTGAACTTTAAAGAATGGCAGAAAGCGTTTACCGATGTGATGGGCA<br>TGGATGAACTGTATAAAGGCGGTAGCGGCGGTAGCCACCATCACCATCACCATTA<br>AA**GCATAGCACAACGATAGCATT**
gDT034	R_1 (M)	**AGCATAGCACAACGATAGCATT**tactagagaaagaggagaaatactaaatgAACaccAGCgaa<br>CTGgaaaccctgattcgcaccattctgagcGAACAGctgaccACCccggcgCAGACCccgGTGcagc<br>cgcagggcAAAGGCattTTTcagAGCGATAGCACCGAAGCGGTGATTAAAGAATTTATG<br>CGCTTTAAAGTGCATATGGAAGGCAGCATGAACGGCCATGAATTTGAAATTGAAG<br>GCGAAGGCGAAGGCCGCCCGTATGAAGGCACCCAGACCGCGAAACTGAAAGTG<br>ACCAAAGGCGGCCCGCTGCCGTTTAGCTGGGATATTCTGAGCCCGCAGTTTATG<br>TATGGCAGCCGCGCGTTTATTAAACATCCGGCGGATATTCCGGATTATTGGAAACA<br>GAGCTTTCCGGAAGGCTTTAAATGGGAACGCGTGATGATTTTTGAAGATGGCGG<br>CACCGTGAGCGTGACCCAGGATACCAGCCTGGAAGATGGCACCCTGATTTATAAA<br>GTGAAACTGCGCGGCGGCAACTTTCCGCCGGATGGCCCGGTGATGCAGAAACG<br>CACCATGGGCTGGGAAGCGAGCACCGAACGCCTGTATCCGGAAGATGTGGTGC<br>TGAAAGGCGATATTAAAATGGCGCTGCGCCTGAAAGATGGCGGCCGCTATCTGG<br>CGGATTTTAAAACCACCTATAAAGCGAAAAAACCGGTGCAGATGCCGGGCGCGTT<br>TAACATTGATCGCAAACTGGATATTACCAGCCATAACGAAGATTATACCGTGGTGG<br>AACAGTATGAACGCAGCGTGGCGCGCCATAGCACCGGCGGCAGCGGCGGCAGC<br>TAA**TCGTTCAGTTTGGCTAACTCAT**

gDT035	B_1 (M)	**TCGTTCAGTTTGGCTAACTCAT**tactagagaaagaggagaaatactaaatgAACaccAGCgaaC<br>TGgaaaccctgattcgcaccattctgagcGAACAGctgaccACCccggcgCAGACCccgGTGcagccg<br>cagggcAAAGGCattTTTcagAGCGTGAGCAAAGGCGAAGAACTGATTAAAGAAAACA<br>TGCATATGAAACTGTATATGGAAGGCACCGTGGATAACCATCATTTTAAATGCACC<br>AGCGAAGGCGAAGGCAAACCGTATGAAGGCACCCAGACCATGCGCATTAAAGTG<br>GTGGAAGGCGGCCCGCTGCCGTTTGCGTTTGATATTCTGGCGACCAGCTTTCTG<br>TATGGCAGCAAAACCTTTATTAACCATACCCAGGGCATTCCGGATTTTTTTAAACA<br>GAGCTTTCCGGAAGGCTTTACCTGGGAACGCGTGACCACCTATGAAGATGGCGG<br>CGTGCTGACCGCGACCCAGGATACCAGCCTGCAGGATGGCTGCCTGATTTATAA<br>CGTGAAAATTCGCGGCGTGAACTTTACCAGCAACGGCCCGGTGATGCAGAAAAA<br>AACCCTGGGCTGGGAAGCGTTTACCGAAACCCTGTATCCGGCGGATGGCGGCC<br>TGGAAGGCCGCAACGATATGGCGCTGAAACTGGTGGGCGGCAGCCATCTGATTG<br>CGAACGCGAAAACCACCTATCGCAGCAAAAAACCGGCGAAAAACCTGAAAATGC<br>CGGGCGTGTATTATGTGGATTATCGCCTGGAACGCATTAAAGAAGCGAACAACGA<br>AACCTATGTGGAACAGCATGAAGTGGCGGTGGCGCGCTATTGCGATCTGCCGAG<br>CAAACTGGGCCATAAACTGAACTAA**ATGAGAGAAGATTTTCAGCCTGA**
gDT036	G_2 (M)	**TTTTTGGGCTAGCGAATTCG**tactagagaaagaggagaaatactaaatgAACaccAGCgaaCTG<br>gaaaccctgattcgcaccattctgagcGAACAGctgaccACCccggcgCAGACCccgGTGcagccgca<br>gggcAAAGGCattTTTcagAGCAACacgAGCgaaCTGgagaccctgatccgcacgattctgtcgGAA<br>CAActgaccACGccggcgCAAACCcctGTGcagcctcagggtAAAGGCattTTCcagAGCGTGA<br>GCAAAGGCGAAGAAGATAACATGGCGAGCCTGCCGGCGACCCATGAACTGCATA<br>TTTTTGGCAGCATTAACGGCGTGGATTTTGATATGGTGGGCCAGGGCACCGGCA<br>ACCCGAACGATGGCTATGAAGAACTGAACCTGAAAAGCACCAAAGGCGATCTGC<br>AGTTTAGCCCGTGGATTCTGGTGCCGCATATTGGCTATGGCTTTCATCAGTATCTG<br>CCGTATCCGGATGGCATGAGCCCGTTTCAGGCGGCGATGGTGGATGGCAGCGG<br>CTATCAGGTGCATCGCACCATGCAGTTTGAAGATGGCGCGAGCCTGACCGTGAA<br>CTATCGCTATACCTATGAAGGCAGCCATATTAAAGGCGAAGCGCAGGTGAAAGGC<br>ACCGGCTTTCCGGCGGATGGCCCGGTGATGACCAACAGCCTGACCGCGGCGGA<br>TTGGTGCCGCAGCAAAAAAACCTATCCGAACGATAAAACCATTATTAGCACCTTTA<br>AATGGAGCTATACCACCGGCAACGGCAAACGCTATCGCAGCACCGCGCGCACCA<br>CCTATACCTTTGCGAAACCGATGGCGGCGAACTATCTGAAAAACCAGCCGATGTA<br>TGTGTTTCGCAAGACCGAACTGAAACATAGCAAAACGGAGCTGAACTTTAAAGAA<br>TGGCAGAAAGCGTTTACCGATGTGATGGGCATGGATGAACTGTATAAAGGCGGTA<br>GCGGCGGTAGCCACCATCACCATCACCATTAA**AGCATAGCACAACGATAGCATT**
gDT037	R_2 (M)	**AGCATAGCACAACGATAGCATT**tactagagaaagaggagaaatactaaatgAACaccAGCgaa<br>CTGgaaaccctgattcgcaccattctgagcGAACAGctgaccACCccggcgCAGACCccgGTGcagc<br>cgcagggcAAAGGCattTTTcagAGCAACacgAGCgaaCTGgagaccctgatccgcacgattctgtcg<br>GAACAActgaccACGccggcgCAAACCcctGTGcagcctcagggtAAAGGCattTTCcagAGCG<br>ATAGCACCGAAGCGGTGATTAAAGAATTTATGCGCTTTAAAGTGCATATGGAAGGC<br>AGCATGAACGGCCATGAATTTGAAATTGAAGGCGAAGGCGAAGGCCGCCCGTAT<br>GAAGGCACCCAGACCGCGAAACTGAAAGTGACCAAAGGCGGCCCGCTGCCGTT<br>TAGCTGGGATATTCTGAGCCCGCAGTTTATGTATGGCAGCCGCGCGTTTATTAAAC<br>ATCCGGCGGATATTCCGGATTATTGGAAACAGAGCTTTCCGGAAGGCTTTAAATG<br>GGAACGCGTGATGATTTTTGAAGATGGCGGCACCGTGAGCGTGACCCAGGATAC<br>CAGCCTGGAAGATGGCACCCTGATTTATAAAGTGAAACTGCGCGGCGGCAACTT<br>TCCGCCGGATGGCCCGGTGATGCAGAAACGCACCATGGGCTGGGAAGCGAGCA<br>CCGAACGCCTGTATCCGGAAGATGTGGTGCTGAAAGGCGATATTAAAATGGCGCT<br>GCGCCTGAAAGATGGCGGCCGCTATCTGGCGGATTTTAAAACCACCTATAAAGCG<br>AAAAAACCGGTGCAGATGCCGGGCGCGTTTAACATTGATCGCAAACTGGATATTA<br>CCAGCCATAACGAAGATTATACCGTGGTGGAACAGTATGAACGCAGCGTGGCGC<br>GCCATAGCACCGGCGGCAGCGGCGGCAGCTAA**TCGTTCAGTTTGGCTAACTCAT**
gDT038	B_2 (M)	**TCGTTCAGTTTGGCTAACTCAT**tactagagaaagaggagaaatactaaatgAACaccAGCgaaC<br>TGgaaaccctgattcgcaccattctgagcGAACAGctgaccACCccggcgCAGACCccgGTGcagccg<br>cagggcAAAGGCattTTTcagAGCAACacgAGCgaaCTGgagaccctgatccgcacgattctgtcgG<br>AACAActgaccACGccggcgCAAACCcctGTGcagcctcagggtAAAGGCattTTCcagAGCGT<br>GAGCAAAGGCGAAGAACTGATTAAAGAAAACATGCATATGAAACTGTATATGGAAG<br>GCACCGTGGATAACCATCATTTTAAATGCACCAGCGAAGGCGAAGGCAAACCGTA<br>TGAAGGCACCCAGACCATGCGCATTAAAGTGGTGGAAGGCGGCCCGCTGCCGT<br>TTGCGTTTGATATTCTGGCGACCAGCTTTCTGTATGGCAGCAAAACCTTTATTAAC<br>CATACCCAGGGCATTCCGGATTTTTTTAAACAGAGCTTTCCGGAAGGCTTTACCT<br>GGGAACGCGTGACCACCTATGAAGATGGCGGCGTGCTGACCGCGACCCAGGAT<br>ACCAGCCTGCAGGATGGCTGCCTGATTTATAACGTGAAAATTCGCGGCGTGAACT<br>TTACCAGCAACGGCCCGGTGATGCAGAAAAAAACCCTGGGCTGGGAAGCGTTTA<br>CCGAAACCCTGTATCCGGCGGATGGCGGCCTGGAAGGCCGCAACGATATGGCG<br>CTGAAACTGGTGGGCGGCAGCCATCTGATTGCGAACGCGAAAACCACCTATCGC

		AGCAAAAAACCGGCGAAAAACCTGAAAATGCCGGGCGTGTATTATGTGGATTATC<br>GCCTGGAACGCATTAAAGAAGCGAACAACGAAACCTATGTGGAACAGCATGAAG<br>TGGCGGTGGCGCGCTATTGCGATCTGCCGAGCAAACTGGGCCATAAACTGAACT<br>AA**ATGAGAGAAGATTTTCAGCCTGA**
gDT039	G_3 (M)	**TTTTTGGGCTAGCGAATTCG**tactagagaaagaggagaaatactaaatgAACaccAGCgaaCTG<br>gaaaccctgattcgcaccattctgagcGAACAGctgaccACCccggcgCAGACCccgGTGcagccgca<br>gggcAAAGGCattTTTcagAGCAACacgAGCgaaCTGgagaccctgatccgcacgattctgtcgGAA<br>CAActgaccACGccggcgCAAACCcctGTGcagcctcagggtAAAGGCattTTCcagAGCAATact<br>AGCgagCTGgagactctgattcgtactattctgtcgGAGCAGctgacgACCccggcaCAGACCccgGT<br>TcagcctcaaggcAAGGGTattTTTcagAGCGTGAGCAAAGGCGAAGAAGATAACATGGC<br>GAGCCTGCCGGCGACCCATGAACTGCATATTTTTGGCAGCATTAACGGCGTGGAT<br>TTTGATATGGTGGGCCAGGGCACCGGCAACCCGAACGATGGCTATGAAGAACTG<br>AACCTGAAAAGCACCAAAGGCGATCTGCAGTTTAGCCCGTGGATTCTGGTGCCG<br>CATATTGGCTATGGCTTTCATCAGTATCTGCCGTATCCGGATGGCATGAGCCCGTT<br>TCAGGCGGCGATGGTGGATGGCAGCGGCTATCAGGTGCATCGCACCATGCAGTT<br>TGAAGATGGCGCGAGCCTGACCGTGAACTATCGCTATACCTATGAAGGCAGCCAT<br>ATTAAAGGCGAAGCGCAGGTGAAAGGCACCGGCTTTCCGGCGGATGGCCCGGT<br>GATGACCAACAGCCTGACCGCGGCGGATTGGTGCCGCAGCAAAAAAACCTATCC<br>GAACGATAAAACCATTATTAGCACCTTTAAATGGAGCTATACCACCGGCAACGGCA<br>AACGCTATCGCAGCACCGCGCGCACCACCTATACCTTTGCGAAACCGATGGCGG<br>CGAACTATCTGAAAAACCAGCCGATGTATGTGTTTCGCAAGACCGAACTGAAACA<br>TAGCAAAACGGAGCTGAACTTTAAAGAATGGCAGAAAGCGTTTACCGATGTGATG<br>GGCATGGATGAACTGTATAAAGGCGGTAGCGGCGGTAGCCACCATCACCATCAC<br>CATTAA**AGCATAGCACAACGATAGCATT**
gDT091	G_1 PduD EP	**TTTTTGGGCTAGCGAATTCG**tactagagaaagaggagaaatactaaATGGAAATTAATGAAA<br>AACTGCTGCGCCAGATTATTGAAGACGTACTGCGCGATATGAAGGGCAGCGATAA<br>ACCGGTCTCGTTTAATGCGCCTGCGGCAAGCACCGCAGTGAGCAAAGGCGAAG<br>AAGATAACATGGCGAGCCTGCCGGCGACCCATGAACTGCATATTTTTGGCAGCAT<br>TAACGGCGTGGATTTTGATATGGTGGGCCAGGGCACCGGCAACCCGAACGATGG<br>CTATGAAGAACTGAACCTGAAAAGCACCAAAGGCGATCTGCAGTTTAGCCCGTG<br>GATTCTGGTGCCGCATATTGGCTATGGCTTTCATCAGTATCTGCCGTATCCGGATG<br>GCATGAGCCCGTTTCAGGCGGCGATGGTGGATGGCAGCGGCTATCAGGTGCAT<br>CGCACCATGCAGTTTGAAGATGGCGCGAGCCTGACCGTGAACTATCGCTATACCT<br>ATGAAGGCAGCCATATTAAAGGCGAAGCGCAGGTGAAAGGCACCGGCTTTCCGG<br>CGGATGGCCCGGTGATGACCAACAGCCTGACCGCGGCGGATTGGTGCCGCAGC<br>AAAAAAACCTATCCGAACGATAAAACCATTATTAGCACCTTTAAATGGAGCTATACC<br>ACCGGCAACGGCAAACGCTATCGCAGCACCGCGCGCACCACCTATACCTTTGCG<br>AAACCGATGGCGGCGAACTATCTGAAAAACCAGCCGATGTATGTGTTTCGCAAGA<br>CCGAACTGAAACATAGCAAAACGGAGCTGAACTTTAAAGAATGGCAGAAAGCGTT<br>TACCGATGTGATGGGCATGGATGAACTGTATAAAGGCGGTAGCGGCGGTAGCCA<br>CCATCACCATCACCATTAA**ATGAGAGAAGATTTTCAGCCTGA**
gDT092	G_1 PduE EP	**TTTTTGGGCTAGCGAATTCG**tactagagaaagaggagaaatactaaATGAATACCGACGCAA<br>TTGAATCGATGGTCCGCGACGTACTGAGCCGCATGAACAGCCTGCAGGGCGATG<br>CGCCGGCAGCGGCGCCTGCGGCAGGCGGCACGAGCCGCGTGAGCAAAGGCG<br>AAGAAGATAACATGGCGAGCCTGCCGGCGACCCATGAACTGCATATTTTTGGCAG<br>CATTAACGGCGTGGATTTTGATATGGTGGGCCAGGGCACCGGCAACCCGAACGA<br>TGGCTATGAAGAACTGAACCTGAAAAGCACCAAAGGCGATCTGCAGTTTAGCCC<br>GTGGATTCTGGTGCCGCATATTGGCTATGGCTTTCATCAGTATCTGCCGTATCCGG<br>ATGGCATGAGCCCGTTTCAGGCGGCGATGGTGGATGGCAGCGGCTATCAGGTG<br>CATCGCACCATGCAGTTTGAAGATGGCGCGAGCCTGACCGTGAACTATCGCTATA<br>CCTATGAAGGCAGCCATATTAAAGGCGAAGCGCAGGTGAAAGGCACCGGCTTTC<br>CGGCGGATGGCCCGGTGATGACCAACAGCCTGACCGCGGCGGATTGGTGCCG<br>CAGCAAAAAAACCTATCCGAACGATAAAACCATTATTAGCACCTTTAAATGGAGCT<br>ATACCACCGGCAACGGCAAACGCTATCGCAGCACCGCGCGCACCACCTATACCT<br>TTGCGAAACCGATGGCGGCGAACTATCTGAAAAACCAGCCGATGTATGTGTTTCG<br>CAAGACCGAACTGAAACATAGCAAAACGGAGCTGAACTTTAAAGAATGGCAGAAA<br>GCGTTTACCGATGTGATGGGCATGGATGAACTGTATAAAGGCGGTAGCGGCGGTA<br>GCCACCATCACCATCACCATTAA**ATGAGAGAAGATTTTCAGCCTGA**
gDT093	G_1 EutC EP	**TTTTTGGGCTAGCGAATTCG**tactagagaaagaggagaaatactaaATGGATCAAAAACAGA<br>TTGAAGAAATTGTACGTAGCGTGATGGCGAGCATGGGCCAGGACGTACCGCAGC<br>CGGCCGCGCCGAGCACGCAGGAAGGCGCAAAGCCGCAGGTGAGCAAAGGCGA<br>AGAAGATAACATGGCGAGCCTGCCGGCGACCCATGAACTGCATATTTTTGGCAGC<br>ATTAACGGCGTGGATTTTGATATGGTGGGCCAGGGCACCGGCAACCCGAACGAT<br>GGCTATGAAGAACTGAACCTGAAAAGCACCAAAGGCGATCTGCAGTTTAGCCCG

		TGGATTCTGGTGCCGCATATTGGCTATGGCTTTCATCAGTATCTGCCGTATCCGGA<br>TGGCATGAGCCCGTTTCAGGCGGCGATGGTGGATGGCAGCGGCTATCAGGTGC<br>ATCGCACCATGCAGTTTGAAGATGGCGCGAGCCTGACCGTGAACTATCGCTATAC<br>CTATGAAGGCAGCCATATTAAAGGCGAAGCGCAGGTGAAAGGCACCGGCTTTCC<br>GGCGGATGGCCCGGTGATGACCAACAGCCTGACCGCGGCGGATTGGTGCCGCA<br>GCAAAAAAACCTATCCGAACGATAAAACCATTATTAGCACCTTTAAATGGAGCTATA<br>CCACCGGCAACGGCAAACGCTATCGCAGCACCGCGCGCACCACCTATACCTTTG<br>CGAAACCGATGGCGGCGAACTATCTGAAAAACCAGCCGATGTATGTGTTTCGCAA<br>GACCGAACTGAAACATAGCAAAACGGAGCTGAACTTTAAAGAATGGCAGAAAGC<br>GTTTACCGATGTGATGGGCATGGATGAACTGTATAAAGGCGGTAGCGGCGGTAGC<br>CACCATCACCATCACCATTAA**ATGAGAGAAGATTTTCAGCCTGA**
gDT094	G_1 CcmN EP	**TTTTTGGGCTAGCGAATTCG**tactagagaaagaggagaaatactaaATGCACCATCACCATC<br>ACCATGGCGGTAGCGTGAGCAAAGGCGAAGAAGATAACATGGCGAGCCTGCCG<br>GCGACCCATGAACTGCATATTTTTGGCAGCATTAACGGCGTGGATTTTGATATGGT<br>GGGCCAGGGCACCGGCAACCCGAACGATGGCTATGAAGAACTGAACCTGAAAA<br>GCACCAAAGGCGATCTGCAGTTTAGCCCGTGGATTCTGGTGCCGCATATTGGCTA<br>TGGCTTTCATCAGTATCTGCCGTATCCGGATGGCATGAGCCCGTTTCAGGCGGC<br>GATGGTGGATGGCAGCGGCTATCAGGTGCATCGCACCATGCAGTTTGAAGATGG<br>CGCGAGCCTGACCGTGAACTATCGCTATACCTATGAAGGCAGCCATATTAAAGGC<br>GAAGCGCAGGTGAAAGGCACCGGCTTTCCGGCGGATGGCCCGGTGATGACCAA<br>CAGCCTGACCGCGGCGGATTGGTGCCGCAGCAAAAAAACCTATCCGAACGATAA<br>AACCATTATTAGCACCTTTAAATGGAGCTATACCACCGGCAACGGCAAACGCTATC<br>GCAGCACCGCGCGCACCACCTATACCTTTGCGAAACCGATGGCGGCGAACTATC<br>TGAAAAACCAGCCGATGTATGTGTTTCGCAAGACCGAACTGAAACATAGCAAAAC<br>GGAGCTGAACTTTAAAGAATGGCAGAAAGCGTTTACCGATGTGATGGGCATGGAT<br>GAACTGTATAAAagcAGCAGCgagCCGgccGGCCGCtcgcctcaaAGCAGCgcaatcgccca<br>cCCGactaaggtctacggcaaggaacagtttCTGCGCatgcgccagagcatgttcCCGgatcgcTAA**ATG<br>AGAGAAGATTTTCAGCCTGA**
gDT115	E2-Crimson	**TTTTTGGGCTAGCGAATTCG**tactagagaaagaggagaaatactaaatgGATAGCACCGAAA<br>ACGTGATTAAACCGTTTATGCGCTTTAAAGTGCATATGGAAGGCAGCGTGAACGG<br>CCATGAATTTGAAATTGAAGGCGTGGGCGAAGGCAAACCGTATGAAGGCACCCA<br>GACCGCGAAACTGCAGGTGACCAAAGGCGGCCCGCTGCCGTTTGCGTGGGATA<br>TTCTGAGCCCGCAGTTTTTTATGGCAGCAAAGCGTATATTAAACATCCGGCGGAT<br>ATTCCGGATTATCTGAAACAGAGCTTTCCGGAAGGCTTTAAATGGGAACGCGTGA<br>TGAACTTTGAAGATGGCGGCGTGGTGACCGTGACCCAGGATAGCAGCCTGCAG<br>GATGGCACCCTGATTTATCATGTGAAATTTATTGGCGTGAACTTTCCGAGCGATGG<br>CCCGGTGATGCAGAAAAAAACCCTGGGCTGGGAACCGAGCACCGAACGCAACT<br>ATCCGCGCGATGGCGTGCTGAAAGGCGAAAACCATATGGCGCTGAAACTGAAAG<br>GCGGCGGCCATTATCTGTGCGAATTTAAAAGCATTTATATGGCGAAAAAACCGGT<br>GAAACTGCCGGGCTATCATTATGTGGATTATAAACTGGATATTACCAGCCATAACG<br>AAGATTATACCGTGGTGGAACAGTATGAACGCGCGGAAGCGCGCCATCATCTGTT<br>TCAGTAA**ATGAGAGAAGATTTTCAGCCTGA**
gDT116	EP-E2-Crimson	**TTTTTGGGCTAGCGAATTCG**tactagagaaagaggagaaatactaaatgAACaccAGCgaaCTG<br>gaaaccctgattcgcaccattctgagcGAACAGctgaccACCccggcgCAGACCccgGTGcagccgca<br>gggcAAAGGCattTTTcagAGCGATAGCACCGAAAACGTGATTAAACCGTTTATGCGC<br>TTTAAAGTGCATATGGAAGGCAGCGTGAACGGCCATGAATTTGAAATTGAAGGCG<br>TGGGCGAAGGCAAACCGTATGAAGGCACCCAGACCGCGAAACTGCAGGTGACC<br>AAAGGCGGCCCGCTGCCGTTTGCGTGGGATATTCTGAGCCCGCAGTTTTTTATG<br>GCAGCAAAGCGTATATTAAACATCCGGCGGATATTCCGGATTATCTGAAACAGAGC<br>TTTCCGGAAGGCTTTAAATGGGAACGCGTGATGAACTTTGAAGATGGCGGCGTG<br>GTGACCGTGACCCAGGATAGCAGCCTGCAGGATGGCACCCTGATTTATCATGTG<br>AAATTTATTGGCGTGAACTTTCCGAGCGATGGCCCGGTGATGCAGAAAAAAACCC<br>TGGGCTGGGAACCGAGCACCGAACGCAACTATCCGCGCGATGGCGTGCTGAAA<br>GGCGAAAACCATATGGCGCTGAAACTGAAAGGCGGCGGCCATTATCTGTGCGAA<br>TTTAAAAGCATTTATATGGCGAAAAAACCGGTGAAACTGCCGGGCTATCATTATGT<br>GGATTATAAACTGGATATTACCAGCCATAACGAAGATTATACCGTGGTGGAACAGT<br>ATGAACGCGCGGAAGCGCGCCATCATCTGTTTCAGTAA**ATGAGAGAAGATTTTC<br>AGCCTGA**
gDT155	G_1 E7R/R11E	**TTTTTGGGCTAGCGAATTCG**tactagagaaagaggagaaatactaaatgAACaccAGCgaaCTG<br>CGCaccctgattGAAaccattctgagcGAACAGctgaccACCccggcgCAGACCccgGTGcagccg<br>cagggcAAAGGCattTTTcagAGCGTGAGCAAAGGCGAAGAAGATAACATGGCGAGC<br>CTGCCGGCGACCCATGAACTGCATATTTTTGGCAGCATTAACGGCGTGGATTTTG<br>ATATGGTGGGCCAGGGCACCGGCAACCCGAACGATGGCTATGAAGAACTGAACC<br>TGAAAAGCACCAAAGGCGATCTGCAGTTTAGCCCGTGGATTCTGGTGCCGCATAT

		TGGCTATGGCTTTCATCAGTATCTGCCGTATCCGGATGGCATGAGCCCGTTTCAG<br>GCGGCGATGGTGGATGGCAGCGGCTATCAGGTGCATCGCACCATGCAGTTTGAA<br>GATGGCGCGAGCCTGACCGTGAACTATCGCTATACCTATGAAGGCAGCCATATTA<br>AAGGCGAAGCGCAGGTGAAAGGCACCGGCTTTCCGGCGGATGGCCCGGTGATG<br>ACCAACAGCCTGACCGCGGCGGATTGGTGCCGCAGCAAAAAAACCTATCCGAAC<br>GATAAAACCATTATTAGCACCTTTAAATGGAGCTATACCACCGGCAACGGCAAACG<br>CTATCGCAGCACCGCGCGCACCACCTATACCTTTGCGAAACCGATGGCGGCGAA<br>CTATCTGAAAAACCAGCCGATGTATGTGTTTCGCAAGACCGAACTGAAACATAGC<br>AAAACGGAGCTGAACTTTAAAGAATGGCAGAAAGCGTTTACCGATGTGATGGGCA<br>TGGATGAACTGTATAAAGGCGGTAGCGGCGGTAGCCACCATCACCATCACCATTA<br>A**ATGAGAGAAGATTTTCAGCCTGA**
